# Comparison of Bowel Preparation Quality for Colonoscopy Between Inpatients and Outpatients: A Single-Center Prospective Observational Study in Lebanon

**DOI:** 10.7759/cureus.98483

**Published:** 2025-12-04

**Authors:** Nayef Alkhalil, Sara Ayoub, Nathalie Nasser, Rola Bou Serhal, Abed AlRaouf Kawtharani, Rola Hussein, Bilal Hotayt

**Affiliations:** 1 Division of Gastroenterology, Department of Medicine, Faculty of Medical Sciences, Lebanese University, Beirut, LBN; 2 Faculty of Medical Sciences, Lebanese University, Beirut, LBN; 3 Division of Research, Modern University for Business and Science (MUBS), Beirut, LBN; 4 Division of Gastroenterology, Al Rassoul Al-Aazam Hospital, Beirut, LBN

**Keywords:** boston bowel preparation score, bowel cleansing, inpatients, smoking, split-dose regimen

## Abstract

Background: Inadequate bowel cleansing is a common challenge among patients undergoing colonoscopy. However, no prior study has specifically investigated whether admission status itself contributes to suboptimal preparation in the Lebanese population.

Objectives: To compare the adequacy of bowel preparation between inpatients and outpatients and to identify independent predictors of poor bowel cleansing.

Methods: This prospective, single-center observational study was conducted at a tertiary care hospital and included colonoscopies performed on both inpatients and outpatients. A total of 217 procedures were analyzed (109 outpatients and 108 inpatients). Bowel preparation quality was evaluated using the Boston Bowel Preparation Scale (BBPS). Univariate and multivariate logistic regression analyses were used to identify factors associated with inadequate bowel preparation.

Results: No statistically significant difference in bowel preparation adequacy was found between inpatients and outpatients. In the overall cohort, two independent predictors of bowel preparation quality were identified. Smoking was significantly associated with inadequate cleansing, with smokers having 62.4% lower odds of achieving good preparation compared to non-smokers (OR = 0.376; p = 0.002). Conversely, the use of a split-dose regimen significantly improved bowel cleansing, increasing the odds of adequate preparation by nearly five times compared to single-dose regimens (OR = 4.96; p = 0.001).

Conclusions: Inpatient status was not associated with inferior bowel preparation compared to outpatient status. Smoking and the type of bowel preparation regimen (split- vs. single-dose) were the most significant predictors of bowel cleansing quality.

## Introduction

Colonoscopy, also referred to as lower gastrointestinal (GI) endoscopy, is a diagnostic and therapeutic procedure that allows direct visualization of the colon and the distal terminal ileum. It is performed using a flexible endoscope equipped with a high-definition camera and accessory channels for insufflation, suction, irrigation, and instrumentation. Colonoscopy plays a central role in diagnosing and managing various GI disorders, including colorectal cancer (CRC), inflammatory bowel disease (IBD), and lower GI bleeding [1,2].

Indications for colonoscopy are generally classified into diagnostic and therapeutic categories. Diagnostic indications include CRC screening, evaluation of unexplained diarrhea, occult or overt GI bleeding, and post-surgical surveillance [2]. For average-risk individuals aged 50-75 years, the United States Preventive Services Task Force recommends screening colonoscopy every 10 years (Grade A recommendation) [3]. Therapeutically, colonoscopy is used for polypectomy, hemostasis, decompression of colonic volvulus, and dilation or stenting of strictures. In many cases, it has replaced or significantly reduced the need for surgical intervention [2].

A successful colonoscopy is highly dependent on adequate bowel preparation. Poor bowel cleansing not only reduces diagnostic accuracy but also increases the risk of missed lesions, prolonged procedure times, and the need for early repeat colonoscopy [4,5]. Studies have shown that high-quality bowel preparation significantly improves the detection rates of adenomas and polyps, enhances mucosal visualization, and shortens procedural duration [4,5]. Therefore, optimizing bowel preparation is a critical step toward ensuring effective and safe colonoscopy outcomes.

To objectively assess bowel preparation quality, several scoring systems have been developed, including the Ottawa Bowel Preparation Scale [6], Chicago Bowel Preparation Scale [7], Harefield Cleansing Scale [8], and the Boston Bowel Preparation Scale (BBPS) [9]. However, their reliability and validity vary [10]. A large systematic review by Parmar et al. concluded that the BBPS is the most thoroughly validated and reliable scoring tool for clinical and research use [11]. The BBPS rates bowel cleanliness across three colon segments (right, transverse, and left) on a 0-3 scale, with a total score ranging from 0 to 9 [9]. According to current guidelines, colonoscopy should be repeated if any segment receives a BBPS score of less than 2 due to inadequate preparation [10].

Numerous factors affect the adequacy of bowel cleansing. These are typically divided into patient-related and procedure-related variables. Older patients often present with multiple comorbidities that impair bowel motility or compliance with preparation instructions. In a study by Romero et al., male gender was identified as a significant predictor of poor bowel preparation [12]. Additionally, smoking has been shown to negatively impact colonic motility and mucus secretion, thus impairing the effectiveness of purgative agents [13].

From a procedural standpoint, the split-dose regimen, in which the bowel preparation solution is divided into two doses administered several hours apart, has demonstrated superior cleansing efficacy compared to single-dose protocols, where the full volume is consumed at once [10]. Likewise, both clear liquid and low-residue diets are frequently used prior to colonoscopy, and studies have shown comparable results in terms of preparation quality [10,14]. Other influencing factors include the type of bowel cleansing agent used, patient education level, adherence to instructions, and hospitalization status [15].

One area of ongoing investigation is the impact of admission status, which is whether a patient is an inpatient or an outpatient, on bowel preparation quality. While some studies suggest outpatients are more likely to achieve adequate preparation, findings have been inconsistent. For example, Parungao et al. reported that outpatients were 2.75 times more likely to achieve adequate bowel preparation compared to inpatients [16]. Similarly, Almadi et al. found significantly better preparation quality among outpatients (OR 0.63; 95% CI, 0.54-0.73) [5]. In contrast, a study by Tantinam et al. found no significant difference between inpatient and outpatient preparation outcomes, highlighting the need for further clarification [17].

Several factors may contribute to inferior bowel preparation among inpatients. These include reduced mobility, higher burden of comorbidities, and physical limitations due to attachment to monitoring equipment or intravenous fluids, which may hinder compliance with preparation protocols [5].

Despite extensive research into colonoscopy outcomes, inadequate bowel preparation remains a significant challenge, affecting up to 25% of procedures [18]. Identifying the factors associated with poor preparation is essential for improving overall procedural success and patient care.

In Lebanon and the broader Arab region, data on bowel preparation quality and colonoscopy outcomes remain limited, leaving an important gap in both national and regional medical literature. This study aims to address this gap by identifying predictors of inadequate bowel preparation, with particular attention to differences between hospitalized and non-hospitalized (ambulatory) patients. By analyzing demographic, clinical, and procedural factors, we seek to better characterize the prevalence of inadequate bowel preparation in our population and determine the factors that contribute to it. In addition, we aim to report the prevalence of common colonic findings and conditions, which remain underreported in Lebanese studies, thereby providing a foundation for future research. Ultimately, our goal is to generate insights that can guide the development of more tailored and effective bowel preparation protocols, reduce the need for repeat procedures, and improve diagnostic accuracy and patient outcomes.

## Materials and methods

We conducted a prospective observational study at the Endoscopy Unit of Al Rassoul Al-Aazam Hospital, a tertiary care center, between September 2024 and March 2025. A total of 218 adult patients, aged 18 years and above, who were scheduled for elective colonoscopy were enrolled. These included both inpatients and outpatients, undergoing colonoscopy for various clinical indications. Patients were enrolled through a 1:1 selection process to ensure balanced representation across relevant clinical and demographic factors. As this was an observational study, randomization and blinding were not applicable to the study design.

Patients were excluded if they were undergoing emergency colonoscopy, had bowel obstruction, severe cognitive impairment, a history of colectomy, or were pregnant at the time of the procedure. The study protocol was approved by Al Rassoul Al-Aazam Hospital - Institutional Review Board (IRB), 8/2024, and written informed consent was obtained from all participants.

Bowel preparation was performed in accordance with widely accepted clinical practice guidelines and in line with the institutional standard of care. Patients received one of two commonly used purgative agents: either four liters of polyethylene glycol-electrolyte solution (PEG-ELS; Fortrans®) or sodium picosulfate with magnesium citrate solution (SPMC, Citrafleet® or Picoprep®). Two different dosing schedules were offered based on shared decision-making between the treating physician and the patient, taking into account the patient’s comorbidities, personal preference, and cost. In the split-dose regimen, the preparation was divided into two parts: half was taken the evening before the colonoscopy, and the other half on the morning of the procedure. In contrast, the single-dose regimen involved administration of the entire preparation on the evening before the colonoscopy.

All patients were instructed to follow a clear liquid diet on the day prior to the colonoscopy. Preparation instructions were explained in detail by the attending gastroenterologist or a gastroenterology fellow. In cases where a family member was present, they were also included in the educational discussion. Additionally, a written instruction handout was provided to reinforce verbal guidance.

After obtaining informed consent, patient data were collected using a structured questionnaire (Appendix 1). Information was gathered directly from the patient and supplemented from the medical record when necessary. The data collected included demographic variables (such as age and gender), admission status (inpatient vs. outpatient), smoking status, past medical history, educational level, indication for colonoscopy, the purgative agent used, the administration regimen (single vs. split dosing), the quality of bowel preparation, whether cecal and ileal intubation was achieved, and colonoscopy findings. Educational level was classified into six categories: illiterate, pre-secondary education (≤ Grade 9 according to the Lebanese educational system), secondary education (Grades 10 to 12 according to the Lebanese educational system), bachelor’s degree, master’s degree, and PhD.

The quality of bowel preparation was assessed using the BBPS, which assigns a score from 0 to 3 to each of the three colonic segments (right, transverse, and left), resulting in a total score ranging from 0 to 9 (Appendix 2) [19]. A total score of 6 or higher was categorized as good preparation, indicating the absence of solid stool with small amounts of clear fluid that were suctioned easily. A score of 3 to 5 was considered fair, corresponding to the presence of collections of semisolid debris that were cleared with difficulty. A score of 2 or less was defined as poor, indicating the presence of solid or semisolid debris that could not be cleared. For the purpose of analysis, inadequate bowel preparation included both fair and poor categories, while adequate preparation included the good category only. This study design has been described in three prior publications [5,20,21]. The BBPS was developed in 2009 by the Gastroenterology Division at Boston University Medical Center, Boston, USA, and is freely available online for public use [9,22,23].

Data entry was performed by medical doctors involved in the study, and all entries were verified by the principal investigators. After data cleaning, the dataset was coded and exported to IBM SPSS Statistics for Windows, Version 26 (Released 2018; IBM Corp., Armonk, New York, United States) for statistical analysis.

Continuous variables were described using means and standard deviations, while categorical variables were presented as frequencies and percentages. Variables such as medical comorbidities, colonoscopy indications, and endoscopic findings were treated as multiple response variables and analyzed accordingly.

Group comparisons (e.g., between inpatients and outpatients or between preparation quality categories) were conducted using the Independent samples t-test, chi-square test, or Fisher’s exact test, depending on the nature and distribution of the variables. To identify independent predictors of bowel preparation quality while adjusting for potential confounders, a binary logistic regression analysis was performed using the Enter method. A p-value of less than 0.05 was considered statistically significant.

## Results

The final study population included 217 patients, among whom one colonoscopy could not be completed due to poor bowel preparation and patient discomfort.

As summarized in Table 1, the mean age of participants was 59 years (standard deviation, SD = 19), and females comprised 55.3% (n = 120) of the population. Smokers accounted for 52.1% (n = 113). Regarding educational level, 13.4% were illiterate, 32.7% of patients had pre-secondary education, 26.7% had completed high school, and 27.2% held a university degree (bachelor’s or higher). In terms of medical history, 35.1% (n = 123) had cardiovascular disease, 25.7% (n = 90) had metabolic disorders, and 15.7% (n = 55) were previously healthy.

**Table 1:** Comparison of demographic and clinical characteristics between outpatients and inpatients ¹ Independent Samples t-test; ² Pearson’s Chi-Square Test; ³ Fisher’s Exact Test. SD: standard deviation; CVD: cardiovascular diseases; HTN: hypertension; HF: heart failure; CAD: coronary artery disease; VHD: valvular heart disease; CKD: chronic kidney disease; ESRD: end-stage renal disease; DM: diabetes mellitus; DLP: dyslipidemia; IBD: inflammatory bowel disease; CVA: cerebro-vascular accident; MS: multiple sclerosis; PD: Parkinson’s disease

Factors		Outpatients		Inpatients		Total		Statistical Test	p-value
		Mean or Frequency	SD or Percentage %	Mean or Frequency	SD or Percentage %	Mean or Frequency	SD or Percentage %		
Age		52	16	67	18	59	19	-6.316¹	<0.001
Gender	Male	42	43.3%	55	56.7%	97	44.7	3.371²	0.066
	Female	67	55.8%	53	44.2%	120	55.3		
Smoker	No	51	49.0%	53	51.0%	104	47.9%	0.114²	0.736
	Yes	58	51.3%	55	48.7%	113	52.1%		
Educational level	Illiterate	5	17.2%	24	82.8%	29	13.4%	42.772³	< 0.001
	Pre-secondary	30	42.3%	41	57.7%	71	32.7%		
	Secondary	25	43.1%	33	56.9%	58	26.7%		
	Bachelor	37	82.2%	8	17.8%	45	20.7%		
	Master	11	84.6%	2	15.4%	13	6.0%		
	PhD	1	100.0%	0	0.0%	1	0.5%		
Number of comorbidities per patient		1.02	1.02	1.7	0.98	1.36	1.05	-5.054¹	<0.001
Comorbidities (less than 3 vs. 3 or more)	Less Than 3 Comorbidities	101	54.6%	84	45.4%	185	85.3%	955.8²	0.002
	3 or More Comorbidities	8	25.0%	24	75.0%	32	14.7%		
Comorbidities	Previously Healthy	43	12.3%	12	3.4%	55	15.7%	90.153²	<0.001
	History of CVD (HTN, HF, CAD, VHD, etc.)	37	10.6%	86	24.6%	123	35.1%		
	History of CKD or ESRD	3	0.9%	14	4.0%	17	4.9%		
	History of Metabolic Disorder (DM, DLP)	35	10.0%	55	15.7%	90	25.7%		
	History of IBD	4	1.1%	2	0.6%	6	1.7%		
	History of Malignancy	4	1.1%	4	1.1%	8	2.3%		
	History of Thyroid Disease	8	2.3%	3	0.9%	11	3.1%		
	History of Psychiatric Disorder	3	0.9%	2	0.6%	5	1.4%		
	History of Neurological Disorder (CVA, MS, PD, etc.)	0	0.0%	2	0.6%	2	0.6%		
	History (Other)	17	4.9%	16	4.6%	33	9.4%		

When comparing inpatients and outpatients, several statistically significant differences were observed. Outpatients were significantly younger, with a mean age of 52 years versus 67 years among inpatients (p < 0.001). Educational level also differed; 82.8% of illiterate patients (n = 24 of 29) were inpatients, while 83.05% of university graduates (n = 49, total = 59) were outpatients, compared to only 16.95% (n = 10) of inpatients. The burden of comorbidities was significantly higher among inpatients, with an average of 1.7 comorbid conditions per patient, compared to 1.02 in outpatients (p < 0.001). Furthermore, patients with three or more comorbidities were significantly more likely to be inpatients (n = 24; 75% of total patients with ≥3 comorbidities), a difference that was also statistically significant (p = 0.002).

The most common comorbidities observed in the study population were cardiovascular diseases (35.1%), metabolic disorders (25.7%), and chronic kidney disease (4.9%). Notably, 15.7% of participants were previously healthy, the majority of whom were outpatients (78.18%). This further supports the observed trend of a higher comorbidity burden among inpatients.

Out of the 217 patients, 59% (n = 128) achieved good-quality bowel preparation, with no significant difference between inpatients (n = 63; 49.2%) and outpatients (n = 65; 50.8%). The remaining 41% (n = 89) had suboptimal preparation, almost equally distributed between inpatients (n = 44; 49.4%) and outpatients (n = 45; 50.6%) (Table 2).

**Table 2:** Comparison of colonoscopy-related factors in outpatients and inpatients ¹ Pearson's Chi Square Test; ² Fisher's Exact Test. SPMC: sodium picosulfate with magnesium citrate solution; PEG-ELS: polyethylene glycol-electrolyte solution; BBPS: Boston Bowel Preparation Scale [9]; IDA: iron-deficiency anemia; CRC: colorectal cancer; GI: gastrointestinal; Sx: symptoms

Factors		Outpatient		Inpatient		Total		Statistical Test	p-value
		Frequency	Percentage (%)	Frequency	Percentage (%)	Frequency	Percentage (%)		
Purgative	SPMC	75	74.3%	26	25.7%	101	46.8%	42.966¹	< 0.001
	PEG-ELS	34	29.6%	81	70.4%	115	53.2%		
Cecal intubation	No	2	40.0%	3	60.0%	5	2.3%	0.224²	0.682
	Yes	107	50.7%	104	49.3%	211	97.7%		
Ileal intubation	No	19	35.8%	34	64.2%	53	24.7%	6.205¹	0.013
	Yes	90	55.6%	72	44.4%	162	75.3%		
Dosing regimen	Single-dose	105	61.4%	66	38.6%	171	78.8%	40.282¹	<0.001
	Split-dose	4	8.7%	42	91.3%	46	21.2%		
BBPS	Poor (≤ 2)	12	42.9%	16	57.1%	28	12.9%	0.746¹	0.689
	Fair (3 to 5)	32	52.5%	29	47.5%	61	28.1%		
	Good (≥ 6)	65	50.8%	63	49.2%	128	59.0%		
Bowel preparation quality	Suboptimal (Poor & Fair)	44	49.4%	45	50.6%	89	41.0%	0.038¹	0.846
	Good	65	50.8%	63	49.2%	128	59.0%		
Colonoscopy indication	Evaluation of Abdominal Pain	61	16.2%	27	7.2%	88	23.4%	112.779²	<0.001
	Evaluation of Anemia or IDA	21	5.6%	58	15.4%	79	21.0%		
	Alteration in Bowel Habits	37	9.8%	25	6.6%	62	16.5%		
	CRC Screening	30	8.0%	15	4.0%	45	12.0%		
	Streptococcus Bovis Bacteremia	0	0.0%	1	0.3%	1	0.3%		
	Evaluation of Abnormal Abdominal Imaging	2	0.5%	13	3.5%	15	4.0%		
	Evaluation for Possible GI Bleeding (melena, hematochezia, etc.)	10	2.7%	29	7.7%	39	10.4%		
	Evaluation of Nonspecific GI Sx (nausea, bloating, etc.)	36	9.6%	4	1.1%	40	10.6%		
	Other	5	1.3%	2	0.5%	7	1.9%		
Colonoscopy findings	Normal	32	12.4%	34	13.2%	66	25.6%	12.505²	0.135
	Colonic Polyps or Tumors	22	8.5%	31	12.0%	53	20.5%		
	Hemorrhoidal Disease	45	17.4%	28	10.9%	73	28.3%		
	Colonic Ulceration	1	0.4%	2	0.8%	3	1.2%		
	Ileal Ulceration	9	3.5%	6	2.3%	15	5.8%		
	Angiodysplasia	2	0.8%	5	1.9%	7	2.7%		
	Diverticular Disease	15	5.8%	12	4.7%	27	10.5%		
	Colitis	0	0.0%	3	1.2%	3	1.2%		
	Other Findings (melanosis coli, anal fissure, etc.)	6	2.3%	5	1.9%	11	4.3%		

In terms of purgative agents, 46.8% (n = 101) received SPMC (Citrafleet®/Picoprep®), the majority of whom were outpatients (n = 75; 74.3%). The remaining 53.2% (n = 115) received PEG-ELS, predominantly among inpatients (n = 81; 70.4%).

Cecal intubation was successfully achieved in 97.7% (n = 211) of patients, with no notable differences between groups. However, ileal intubation was significantly more successful in outpatients (n = 90; 55.6%).

Regarding the preparation regimen, 78.8% (n = 171) of patients received a single-dose regimen, representing the majority of both outpatients (n = 105) and inpatients (n = 66). The split-dose regimen was used in 21.2% (n = 46) of cases and was more commonly implemented in inpatients (n = 42; 91.3%), likely due to closer monitoring and staff-administered preparation (Table 2).

Patients often presented with multiple indications for colonoscopy. The four most common were evaluation of persistent abdominal pain (n = 88; 23.4%); evaluation of anemia or iron-deficiency anemia (IDA) (n = 79; 21%); altered bowel habits, including chronic constipation or diarrhea (n = 62; 16.5%); and CRC screening (n = 45; 12%)

The distribution of indications differed significantly between inpatients and outpatients (p < 0.001). Among outpatients, the most common indications were evaluation of abdominal pain (n = 61; 16.2%) and altered bowel habits (n = 37; 9.8%). In contrast, inpatients were more frequently referred for evaluation of anemia (n = 58; 15.4%) and GI bleeding (n = 29; 7.7%). Notably, CRC screening was more commonly performed in outpatients (n = 30; 8%) compared to inpatients (n = 15; 4%), as shown in Table 2.

Among colonoscopy findings, the most frequently observed condition was hemorrhoidal disease (28.3%), followed by normal colonoscopy results (25.6%), colonic polyps or tumors (20.5%), diverticular disease (10.5%), ileal ulceration (5.8%), and angiodysplasia, which constituted 2.7% of total endoscopic findings (Table 2).

Univariate analysis was conducted to explore potential factors influencing the quality of bowel preparation (Table 3). The results indicated that non-smokers had a significantly higher rate of good-quality colonoscopies compared to smokers (67.3% vs. 51.3%; p = 0.017), as shown in Table 3. More than two-thirds of colonoscopies performed on non-smokers were of good quality (67.3%), whereas almost half of the procedures in smokers were suboptimal (n = 55; 48.7%, p-value = 0.017).

**Table 3:** Factors affecting bowel preparation quality (univariate analysis) ¹ Independent Samples t-test; ² Pearson's Chi Square Test; ³ Fisher's Exact Test. SD: standard deviation; SPMC: sodium picosulfate with magnesium citrate solution; PEG-ELS: polyethylene glycol-electrolyte solution; IDA: iron-deficiency anemia; CRC: colorectal cancer; GI: gastrointestinal; Sx: symptoms

Factors		Suboptimal or Good Quality				Statistical Test	p-value
		Suboptimal		Good			
		Mean or Frequency	SD or Percentage (%)	Mean or Frequency	SD or Percentage (%)		
Age		62	18	57	19	1.663¹	0.09
Number of comorbidities per patient		1.47	1.02	1.28	1.07	1.313¹	0.19
Gender	Male	41	42.3%	56	57.7%	0.114²	0.736
	Female	48	40.0%	72	60.0%		
Smoker	No	34	32.7%	70	67.3%	5.717²	0.017
	Yes	55	48.7%	58	51.3%		
Educational level	Illiterate	18	62.1%	11	37.9%	8.747³	0.098
	Pre-secondary	25	35.2%	46	64.8%		
	Secondary	20	34.5%	38	65.5%		
	Bachelor	19	42.2%	26	57.8%		
	Master	6	46.2%	7	53.8%		
	PhD	1	100.0%	0	0.0%		
Purgative	SPMC	41	40.6%	60	59.4%	0.002²	0.976
	PEG-ELS	47	40.9%	68	59.1%		
Dosing regimen	Single-dose	78	45.6%	93	54.4%	7.056²	0.008
	Split-dose	11	23.9%	35	76.1%		
Admission status	Outpatient	44	40.4%	65	59.6%	0.038²	0.846
	Inpatient	45	41.7%	63	58.3%		
Comorbidities (less than 3 versus 3 or more)	Less Than 3 Comorbidities	76	41.1%	109	58.9%	0.002²	0.961
	3 or More Comorbidities	13	40.6%	19	59.4%		
Colonoscopy indication	Evaluation of Abdominal Pain	27	7.2%	61	16.2%	7.788³	0.326
	Evaluation of Anemia or IDA	31	8.2%	48	12.8%		
	Alteration in Bowel Habits	23	6.1%	39	10.4%		
	CRC Screening	20	5.3%	25	6.6%		
	Streptococcus Bovis Bacteremia	0	0.0%	1	0.3%		
	Evaluation of Abnormal Abdominal Imaging	8	2.1%	7	1.9%		
	Evaluation for Possible GI Bleeding (melena, hematochezia, etc.)	16	4.3%	23	6.1%		
	Evaluation of Nonspecific GI Sx (nausea, bloating, etc.)	16	4.3%	24	6.4%		
	Other	4	1.1%	3	0.8%		

In terms of the bowel preparation regimen, 76.1% (n = 35) of patients who received a split-dose regimen had good bowel preparation, compared to 54.4% (n = 93) in the single-dose group (p = 0.008).

Although age and educational level did not show statistically significant differences in bowel preparation adequacy, some trends were observed. The mean age of patients with suboptimal preparation was higher (62 years) compared to those with adequate preparation (57 years). Additionally, the proportion of educated patients was consistently higher in the group with good preparation, while 62% of illiterate patients had inadequate preparation.

Other factors, including gender, number of comorbidities, type of bowel preparation, and admission status, did not show a significant association with the quality of bowel preparation (Table 3).

A sub-analysis was conducted to assess the impact of the bowel preparation regimen (single-dose vs. split-dose) on colonoscopy quality, stratifying patients into inpatients and outpatients (Table 4). A statistically significant difference was observed only among inpatients (p = 0.009), where a higher proportion of good-quality colonoscopies was noted in those receiving the split-dose regimen (n = 31; 73.8%) compared to the single-dose group (n = 32; 48.5%).

**Table 4:** Subgroup analysis of bowel preparation quality by dosing regimen and admission status ¹ Fisher's Exact Test; ² Pearson's Chi Square Test.

Admission Status	Dosing Regimen	Quality				Statistical Test	p-value
		Suboptimal		Good			
		Frequency	Percentage %	Frequency	Percentage %		
Outpatients	Single-dose	44	41.90%	61	58.10%	2.811¹	0.146
	Split-dose	0	0.00%	4	100%		
Inpatients	Single-dose	34	51.50%	32	48.50%	6.773²	0.009
	Split-dose	11	26.20%	31	73.80%		

Only four outpatients in the study received a split-dose regimen, and all four had good-quality colonoscopies. However, the comparison group (single-dose outpatients) included 105 patients.

A logistic regression analysis was performed to assess the independent effect of various factors on colonoscopy quality while controlling for potential confounders. The model included the following variables: age, number of comorbidities per patient, gender, smoking status, type of purgative, admission status (inpatient), dosing regimen (split-dose), and educational level (Table 5). The analysis used the enter method and included 216 patients (excluding one patient with an incomplete colonoscopy).

**Table 5:** Logistic regression analysis of predictors of bowel preparation quality Variables entered in the model: age, number of comorbidities per patient, gender, smoking status, type of purgative, admission status, dosing regimen, and educational level. Method = Enter. N = 216. Nagelkerke R² = 18.7%. PEG-ELS: polyethylene glycol-electrolyte solution

Predictors	Odds Ratio (OR)	95% Confidence Interval for OR		p-value
		Lower limit	Upper limit	
Age	0.979	0.954	1.004	0.094
Number of comorbidities per patient	0.943	0.665	1.336	0.740
Gender (Female)	0.998	0.533	1.867	0.994
Smoker (Yes)	0.376	0.203	0.696	0.002
Purgative (PEG-ELS)	1.159	0.569	2.362	0.684
Admission status (Inpatient)	0.666	0.318	1.395	0.281
Dosing regimen (Split-dose)	4.960	1.967	12.504	0.001
Educational level				0.456
Pre-secondary	2.303	0.837	6.337	0.106
Secondary	1.781	0.563	5.629	0.326
Bachelor	1.159	0.324	4.147	0.821
Master	1.040	0.217	4.991	0.961
PhD	0.000	0.000		1.000
Constant	5.214			0.116

The model demonstrated a Nagelkerke R² of 18.7%, indicating that the included variables explained a modest proportion of the variance in colonoscopy quality.

After adjusting for all other variables, smoking was associated with a significantly lower likelihood of a good-quality colonoscopy, with smokers having 62.4% lower odds compared to non-smokers (OR = 0.376; p = 0.002); receiving a split-dose regimen significantly increased the odds of good bowel preparation by nearly five times compared to the single-dose regimen (OR = 4.96; p = 0.001). These results are summarized in Table 5.

## Discussion

A high-quality bowel preparation is essential for colonoscopy to achieve its full diagnostic and therapeutic potential. Adequate bowel cleansing is associated with improved adenoma detection rates, reduced need for repeat procedures, and greater patient satisfaction [5]. Recognizing the importance of this step, the U.S. Multi-Society Task Force on Colorectal Cancer released updated guidelines in March 2025, emphasizing evidence-based strategies to optimize bowel cleansing protocols for colonoscopy [10].

Bowel preparation typically involves the ingestion of a cleansing solution and large volumes of fluid, leading to frequent trips to the restroom. This process can be particularly challenging for hospitalized patients, who may be connected to monitoring equipment, intravenous lines, or restricted by shared or inaccessible restroom facilities in crowded emergency departments or inpatient wards. Intuitively, one might expect that bowel preparation quality would be lower in inpatients compared to outpatients.

However, our study did not find a statistically significant difference in bowel preparation quality between inpatient and outpatient settings (p = 0.846). These findings are consistent with those of Rotondano et al., who conducted a large prospective multicenter study across 25 regional endoscopy units involving 3,276 colonoscopies (2,178 outpatients and 1,098 inpatients), and also reported no significant difference between the two settings [24]. Similarly, Tantinam et al. found no meaningful variation in preparation quality between inpatient and outpatient groups [17].

In contrast, Almadi et al., in a retrospective study involving 2,999 patients, reported significantly better bowel preparation among outpatients (OR = 0.63; 95% CI, 0.54-0.73), suggesting that the clinical setting may influence outcomes in certain contexts [5]. However, their analysis did not account for the dosing regimen, which could confound the results. Additionally, the retrospective design introduces limitations such as potential documentation bias and unmeasured confounding, which should be considered when interpreting their findings.

In our study, inpatients were significantly older (mean age: 67 vs. 52 years, p < 0.001), had a higher burden of comorbidities (mean: 1.7 vs. 1.02, p < 0.001), and were less educated, with 82.8% of illiterate patients belonging to the inpatient group. These demographic patterns are consistent with findings from previous studies [5,17]. Despite these differences, none of these factors were independently associated with bowel preparation quality. Similar results have been reported in other studies [17], suggesting that individual patient characteristics may play a limited role in determining bowel prep adequacy when effective medical care systems are in place.

We believe this reinforces the notion that a clinical setting alone does not dictate preparation quality. Despite inpatients being older and having a higher burden of illness, their close monitoring and adherence to preparation protocols under medical supervision may have compensated for their increased risk of poor bowel preparation. Similarly, although outpatients were generally more educated, educational level was not a significant predictor of bowel preparation quality in our analysis. This could be due to our relatively small sample size or the effect of medical team guidance overriding the role of education.

With regard to independent predictors of bowel preparation quality, our study identified two significant factors: smoking status and the bowel purgative administration regimen. Smoking was associated with a 62.4% reduction in the odds of achieving adequate bowel preparation (OR = 0.376; p = 0.002). In contrast, receiving a split-dose regimen was linked to a nearly fivefold increase in the likelihood of good bowel preparation (OR = 4.96; p = 0.001). These findings are consistent with those reported in the existing literature [25,26], further reinforcing the importance of both patient behavior and regimen timing in influencing preparation outcomes.

Regarding the type of bowel preparation used, outpatients were more commonly prescribed SPMC, while inpatients more frequently received PEG-ELS. This difference is likely driven by the patients’ underlying medical conditions and the safety profiles of these preparations.

SPMC is generally better tolerated and more palatable, making it a preferred choice for healthier individuals. However, it carries a higher risk of fluid shifts and electrolyte disturbances, which can be problematic for patients with comorbidities such as heart failure, liver disease, or renal impairment, conditions more prevalent among inpatients. In contrast, PEG-ELS, although less palatable, is safer for patients with these comorbidities and is therefore the recommended option in higher-risk populations [10].

Despite international guidelines recommending split-dose regimens for optimal bowel cleansing, the majority of patients in our study received a single-dose preparation [10]. This likely reflects common practice among gastroenterologists in Lebanon, where single-dose regimens are often preferred to enhance patient compliance, particularly for early morning procedures, as they eliminate the need for patients to wake up during the night to complete the preparation. Interestingly, split-dose regimens were more frequently administered to certain inpatients, possibly indicating a targeted effort by physicians to improve preparation quality in higher-risk patients.

Our study found that 59% of patients achieved good bowel preparation. While similar rates have been reported in other studies [5], this remains below the ≥90% target recommended by international guidelines [10]. This suboptimal outcome is likely linked to the widespread use of single-dose regimens in Lebanon, emphasizing the need to update local practices in line with current evidence-based recommendations. In a predominantly private healthcare system where patient preferences often influence clinical decisions, enhancing adherence to best practices may require a multifaceted approach combining physician education, increased patient awareness, and greater governmental involvement in the implementation and enforcement of clinical guidelines.

In addition to assessing bowel preparation quality, our study explored differences in colonoscopy indications between inpatient and outpatient populations. The distribution of indications varied significantly between the two groups (p < 0.001). Outpatients were more often referred for non-urgent reasons such as abdominal pain (16.2%) and altered bowel habits (9.8%), while inpatients were more frequently evaluated for anemia (15.4%) and GI bleeding (7.7%), reflecting the acuity of their clinical presentations. As expected, CRC screening was more common among outpatients (8%) than inpatients (4%), consistent with its non-urgent nature. Similar patterns in indication distribution have been reported in previous studies [5].

Colonoscopy findings in our population provide useful baseline data in a region where such information is scarce. The most common findings were hemorrhoidal disease (28.3%), normal results (25.6%), colonic polyps or tumors (20.5%), diverticular disease (10.5%), ileal ulceration (5.8%), and angiodysplasia (2.7%). These findings may serve as a reference for future studies in the Lebanese population and contribute to improving local clinical practice and screening strategies.

To our knowledge, this is the first study conducted in Lebanon to compare bowel preparation quality between inpatients and outpatients while also identifying contributing factors. Notably, as far as we have determined, no previous studies have examined educational level as a variable in this context, making our research unique in exploring its potential influence on preparation quality. The use of the validated BBPS adds robustness and objectivity to our assessment. Our study population represented a wide range of ages and socioeconomic backgrounds, enhancing the generalizability of the findings to the broader Lebanese population and healthcare setting. Moreover, the results are consistent with internationally published literature, further supporting their relevance to routine clinical practice.

However, several limitations should be acknowledged. First, this was a single-center study, which may limit the generalizability of the findings. Second, the relatively small sample size may have reduced the statistical power to detect certain associations; non-parametric tests were employed where appropriate to mitigate this limitation. Third, there was an imbalance in the number of patients receiving single-dose versus split-dose regimens, along with variability in the choice of bowel preparation agents. These differences stem from the observational design, which reflected real-world physician practices rather than standardized protocols. Finally, some potentially relevant patient-related factors, such as a history of chronic constipation and opioid use, were not consistently captured and may have influenced bowel preparation quality.

Although Fisher’s exact test was used to account for small cell sizes, the analysis among outpatients may have been underpowered. Notably, only four outpatients received the split-dose regimen, and all had good-quality colonoscopies. In contrast, the comparison group, outpatients who received the single-dose regimen, included 105 patients. This substantial disparity suggests that a larger sample size would be required to determine whether any meaningful differences exist between dosing strategies in this subgroup.

These limitations may have influenced the observed outcomes and should be addressed in future research. A randomized interventional study design would allow for greater control of confounding variables and provide more definitive conclusions.

## Conclusions

In conclusion, this study offers valuable insight into bowel preparation quality among inpatients and outpatients undergoing colonoscopy in Lebanon. Although no significant difference in preparation quality was observed between the two groups overall, the findings highlight the clear advantage of split-dose regimens, particularly among hospitalized patients, in enhancing bowel quality and potentially reducing the need for repeat procedures. Additionally, smoking emerged as a strong negative predictor of preparation adequacy, emphasizing the importance of addressing modifiable patient behaviors alongside procedural strategies.

Despite its limitations, this study provides a useful reference for improving colonoscopy preparation practices in Lebanon. Future research involving larger, multicenter cohorts is needed to confirm these results and to guide the development of tailored, evidence-based preparation protocols suited to different patient populations.

## Figures and Tables

**Figure 1:**
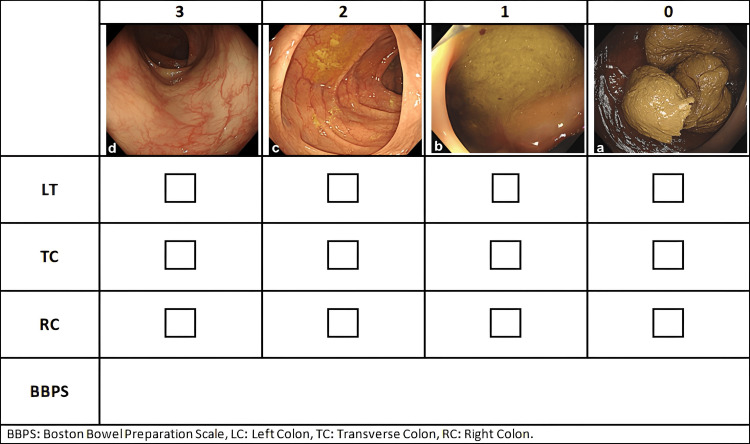
Boston Bowel Preparation Scale (BBPS) Adapted from [19]. This is an open-access article licensed under a Creative Commons Attribution 4.0 International License (CC BY 4.0), which permits unrestricted use, distribution, and reproduction in any medium, provided the original work is properly cited. Each segment is scored as follows: 0 = unprepared colon segment with mucosa not seen due to solid stool that cannot be cleared; 1 = portion of mucosa of the colon segment seen, but other areas of the colon segment not well seen due to staining, residual stool, and/or opaque liquid; 2 = minor amount of residual staining, small fragments of stool and/or opaque liquid, but the mucosa of the colon segment is seen well; 3 = entire mucosa of colon segment seen well with no residual staining, small fragments of stool or opaque liquid.

## Appendices

Appendix 1 

**Table 6:** Study questionnaire

“Comparison of Bowel Preparation Quality for Colonoscopy Between Inpatients and Outpatients: A Single-Center Prospective Observational Study in Lebanon”					
Informed Consent		الموافقة المستنيرة			
Dear participant, We'd like to ask you to participate in our study, as your contribution is invaluable to our research. We want you to know that all information provided will be kept strictly confidential and used solely for research purposes. Your anonymity and privacy are of utmost importance to us. By agreeing to participate, you agree to use your responses for this study. Your participation is voluntary, and you may withdraw at any time if you don't mind. Thank you for considering this invitation to contribute to our research efforts.		عزيزي المشارك، نود أن نطلب منك المشاركة في دراستنا، حيث أن مساهمتك مهمة جدا لبحثنا. نريد منك أن تعلم أن جميع المعلومات المقدمة ستظل سرية للغاية وستستخدم فقط لأغراض البحث. إن عدم الكشف عن هويتك وخصوصيتك لهما أهمية قصوى بالنسبة لنا. بموافقتك على المشاركة، فإنك توافق على استخدام إجاباتك لهذه الدراسة. إن مشاركتك طوعية، ويمكنك الانسحاب في أي وقت. شكرًا لك للإطلاع على هذه الدعوة والمساهمة في جهودنا البحثية.			
Agree	I don’t agree	غير موافق		أوافق	
Dear Researcher, Please circle the appropriate response(s), and record any additional or unlisted information in the space provided or under the "Other" section.					
Patient number					
Gender		Male		Female	
Age (in years)					
Colonoscopy Date					
Smoker?		Yes		No	
Educational Level		Illiterate		Pre-Secondary	
		Secondary		Bachelor’s degree	
		Master’s degree		PhD	
Past Medical History		Previously healthy			
		Other:			
Admission Status		Inpatient		Outpatient	
Colonoscopy Indication		Abdominal pain		CRC Screening	
		Alteration in bowel habits		Evaluation of anemia or IDA	
		Melena		Hematochezia	
		Abnormal abdominal imaging		Bloating	
		Other:			
Purgative		PEG-ELS (Fortranse®)		SPMC (Citrafleet® or Picoprep®)	
Dosing Regimen		Single-dose		Split-dose	
Quality of Bowel Preparation (refer to BBPS sheet)		Poor (≤ 2)	Fair (3 to 5)		Good (≥ 6)
Cecal Intubation		Yes		No	
Ileal Intubation		Yes		No	
Colonoscopy Findings		Normal colonoscopy			
		Other:			

Appendix 2
